# Characterization of the Dispersal of Non-Domiciliated *Triatoma dimidiata* through the Selection of Spatially Explicit Models

**DOI:** 10.1371/journal.pntd.0000777

**Published:** 2010-08-03

**Authors:** Corentin Barbu, Eric Dumonteil, Sébastien Gourbière

**Affiliations:** 1 UMR 5244 CNRS/UPVD/EPHE, ‘Biologie et Ecologie Tropicale et Méditerranéenne’, Université de Perpignan Via Domitia, Perpignan, France; 2 Laboratorio de Parasitología, Centro de Investigaciones Regionales “Dr. Hideyo Noguchi”, Universidad Autónoma de Yucatán, Mérida, Yucatan, Mexico; 3 Department of Tropical Medicine, School of Public Health and Tropical Medicine, Tulane University, New Orleans, Louisiana, United States of America; 4 Centre for the Study of Evolution, School of Biological Sciences, University of Sussex, Brighton, United Kingdom; Universidad de Buenos Aires, Argentina

## Abstract

**Background:**

Chagas disease is a major parasitic disease in Latin America, prevented in part by vector control programs that reduce domestic populations of triatomines. However, the design of control strategies adapted to non-domiciliated vectors, such as *Triatoma dimidiata*, remains a challenge because it requires an accurate description of their spatio-temporal distributions, and a proper understanding of the underlying dispersal processes.

**Methodology/Principal Findings:**

We combined extensive spatio-temporal data sets describing house infestation dynamics by *T. dimidiata* within a village, and spatially explicit population dynamics models in a selection model approach. Several models were implemented to provide theoretical predictions under different hypotheses on the origin of the dispersers and their dispersal characteristics, which we compared with the spatio-temporal pattern of infestation observed in the field. The best models fitted the dynamic of infestation described by a one year time-series, and also predicted with a very good accuracy the infestation process observed during a second replicate one year time-series. The parameterized models gave key insights into the dispersal of these vectors. i) About 55% of the triatomines infesting houses came from the peridomestic habitat, the rest corresponding to immigration from the sylvatic habitat, ii) dispersing triatomines were 5–15 times more attracted by houses than by peridomestic area, and iii) the moving individuals spread on average over rather small distances, typically 40–60 m/15 days.

**Conclusion/Significance:**

Since these dispersal characteristics are associated with much higher abundance of insects in the periphery of the village, we discuss the possibility that spatially targeted interventions allow for optimizing the efficacy of vector control activities within villages. Such optimization could prove very useful in the context of limited resources devoted to vector control.

## Author Summary

Chagas disease is one of the most important neglected diseases in Latin America. Although insecticides have been successfully sprayed to control domiciliated vector populations, this strategy has proven to be ineffective in areas where non-domiciliated vectors immigrating from peridomestic or sylvatic ecotopes can (re-)infest houses. The development of strategies for the control of non-domiciliated vectors has thus been identified by the World Health Organization as a major challenge. Such development primarily requires a description of the spatio-temporal dynamics of infestation by these vectors, and a good understanding of their dispersal. We combined for the first time extensive spatio-temporal data sets describing house infestation dynamics by *Triatoma dimidiata* inside one village, and spatially explicit population dynamics models. The models fitted and predicted remarkably the observed infestation dynamics. They thus provided both key insights into the dispersal of *T. dimidiata* in this area, and a suitable mathematical background to evaluate the efficacy of various control strategies. Interestingly, the observed and modelled patterns of infestation suggest that interventions could focus on the periphery of the village, where there is the highest risk of transmission. Such spatial optimization may allow for reducing the cost of control, compensating for repeated interventions necessary for non-domiciliated vectors.

## Introduction

Chagas disease is caused by the protozoan parasite *Trypanosoma cruzi* and transmitted primarily via hematophagous insects of the Triatominae subfamily. It is the most important vector-borne disease in Latin America, with 8 to 15 million people infected with *T. cruzi*, and about 28 million are at risk of infection. About 41,000 persons become infected every year, and the disease causes 12,500 deaths per year [1], [2].

The elimination of domestic populations of triatomines by insecticide spraying and housing improvement has been the main objective of vector control programs in many countries, and these have led to a large reduction in house infestation by triatomines and a reduction in vector-borne transmission [3], [4]. In spite of this success, house (re-)infestation by non-domiciliated triatomine vectors remains a key challenge for the sustainability of vector control and further reduction of Chagas disease burden [2], [4]–[7]. Indeed, these triatomines are able to disperse from peridomestic and/or sylvatic sites to occasionally infest or re-infest houses. In these conditions, conventional insecticide spraying is of limited efficacy, and control strategies have to be adapted accordingly [8]–[10].

A well-characterized example of such non-domiciliated vector, is *T. dimidiata* from the Yucatan peninsula, Mexico, which is comprised of different taxonomic groups [11]–[13]. Field collections show that adult *T. dimidiata* transiently infest houses on a seasonal basis, during the months of March–July [14]–[16]. Analysis of gene flow between habitats and matrix models confirmed that house infestation is associated with the seasonal dispersal of bugs from the peridomestic and/or sylvatic environment [10], [17], [18]. Triatomine reproduction in the domestic habitat seems to play a negligible role in this process [10], [18]. These data allowed us to start evaluating conventional and novel vector control strategies by using a simple matrix model simulating the observed seasonal immigration of vectors into houses [10]. However, further optimization of vector control requires additional aspects of triatomine dispersal to be better understood and modelled, as they have potential implications on the type and efficacy of interventions that may be designed.

First, the respective contribution of sylvatic and peridomestic bugs to house infestation remains unclear. On one hand, spatio-temporal analysis of house infestation dynamics suggests a sustained influx of sylvatic bugs resulting in a much higher infestation level in houses located in close proximity of sylvatic areas [19], [20]. On the other hand, peridomiciles are known to be colonized [8], and may thus be an important source of infestation, and population genetics indicates the presence of gene flow from both peridomestic and sylvatic habitats [17].

Second, surprisingly little is known on triatomine dispersal, although it is a major process underlying the spatio-temporal patterns of infestation. Flight initiation rates appear very variable, ranging from 5% to over 60% of insects taking off per night, depending on the species, as well as on sex and nutritional status [21]–[23]. Similarly, dispersal distances are not well established. Field tracking studies suggest that *T. infestans* and *T. sordida* are able to fly over distances of 100–200 m, but some individuals may fly up to 1 km [21], [24]. Population redistribution studies gave contradictory results on such long-range dispersal potential of *T. infestans* as the latter was either apparent in the spatial population pattern [25] or not [26]. Assuming a sylvatic origin of *T. dimidiata*, spatial analysis of field collections suggests an average dispersal distance around 100 m for this species, although this varied according to sex and *T. cruzi* infection status [19].

Finally, it is unclear if bug dispersal and house infestation are random events that do not correlate with domestic features, or if houses are particularly attracting dispersing bugs. Indeed, most triatomine species are thought to be attracted by artificial light [27]–[29], but little is known on *T. dimidiata* stimuli for dispersal [15].

In the present work, we combine an extensive spatio-temporal data set describing house infestation dynamics by *T. dimidiata* within a village, and spatial population dynamics models in a selection model approach [30], [31] to improve our understanding of non-domiciliated vectors dispersal by specifically addressing these issues. This approach typically allows to make inference about unobserved processes (here dispersal) based on comparisons between observed patterns (here of village infestation) and theoretical predictions made under various hypotheses about the unobserved processes. We developed spatially explicit population dynamics models which allow us to make inference on spatio-temporal patterns emerging from the interplay between local birth and death processes described in each cell of a grid representing a realistic landscape, and dispersal on the grid [32]. Several of such models were implemented to provide theoretical predictions under different hypotheses on the origin of the dispersers and their dispersal characteristics, which we compared with the spatio-temporal pattern of infestation observed in the field.

## Materials and Methods

### General overview

We used a selection model approach [30], [31] to make inference about the dispersal processes based on the observed patterns of house infestation within a village, by following four steps. First, we used field data from a typical rural village in the Yucatan peninsula, Mexico representing the spatio-temporal pattern of house infestation over a one year study period. Second, different hypotheses about the origin and dispersal abilities of the bugs were formulated and implemented in spatially explicit models describing the vector population dynamic on a grid representing the village and its surroundings. The hypotheses that we examined were related to 1) the relative contribution of sylvatic and peridomestic insects to house infestation, 2) the departure probability from different habitats, 3) the distribution of dispersal distances, and 4) the propensity of bugs to move toward houses. The models produced different spatio-temporal patterns of expected bug abundance in each house of the village, which, in a third step, were fitted to the field data by seeking the set of model parameters that maximized the likelihood of the data set. The predictive capabilities of the parameterized models were then tested on a replicate data set, which corresponded to the observed variations in vector abundance inside houses of the same village during a second year of study. Finally, the parameterized models and their outputs were compared using Akaike Information Criteria (AIC) [33], which gives the relative strength of support of the data for the models, and the corresponding hypotheses, while accounting for both the quality of the fit and the parsimony of the description of the processes.

### Field collections and maps

The spatio-temporal pattern of house infestation was observed in the rural village of Teya, Yucatan, Mexico over a two-year period from August 2006 to October 2008 [19]. The village has 1,966 inhabitants distributed in 518 houses (INEGI 2005 population census). All houses were identified and geo-referenced with a hand held global positioning system (GPS). Insects were collected by a standardized methodology based on community participation. Such methodology has been found highly reliable for entomologic surveys and more sensitive than manual collections where bugs are transiently present and/or in very low density in the domestic habitat [14], [16]. For this, workshops and individual visits to households were organized in the village, to provide information on Chagas disease and the vector. Households were then instructed to collect triatomines found inside their house in plastic vials/bags labelled with their name, address and date of capture, and deposit them at the local Health Center of their village, where all collected bugs were stored under the supervision of health personnel. Bugs were gathered from the local Health Centers during regular visits to the village, every 2–3 weeks. Participating families provided oral consent prior to their participation, as written consent was waived because the study involved no procedures for which written consent is normally required outside of the research context. Consent was logged in field notebooks. All procedures, including the use of oral consent, were approved by the Institutional Bioethics committee of the Regional Research Center “Dr. Hideyo Noguchi”, Universidad Autonoma de Yucatan. Coordinates of all inhabited houses from the villages as well as of triatomine collection sites were imported into a geographic information system (GIS) database in ArcView 3.2 (Environmental Systems Research Institute, Redlands, CA, USA) for analysis. A Google Earth satellite image of the village was imported into the GIS database to provide background landscape information. Village boundaries with the surrounding sylvatic area were drawn based on the satellite images and field observations [19]. A schematic grid map of the village was derived from the satellite image and the GPS locations of the houses, to produce maps of observed triatomine abundance in the houses over 2 weeks intervals and compare these with the model outputs (see below for details on the grid).

### Spatially explicit sources-sink modelling

Our models were based on a grid representing the studied village, and allowed to calculate the temporal variations in the number of bugs in each cell of the grid (thereafter referred to as ‘state variables’) according to ‘local’ rules, describing birth and death processes within cells, and ‘dispersal’ rules which allow coupling the cells.

#### A GIS-based spatially realistic landscape

**Figure 1:**
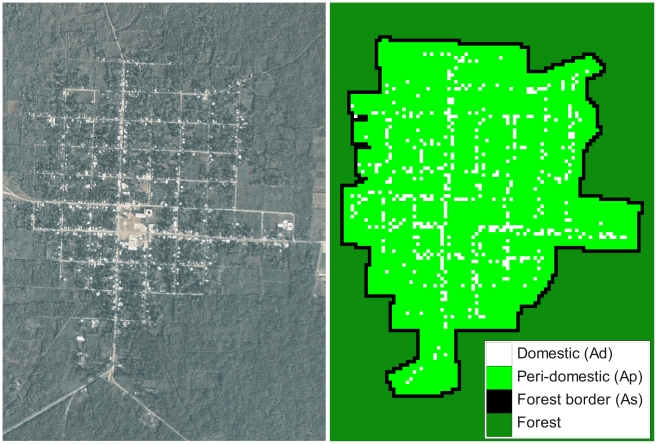
Satellite image and grid of the village of Teya, Yucatan, Mexico. The grid is 88×104 pixels between forest border cells and was derived from the satellite image and the GPS coordinates of all houses. Each cell of the grid is associated with one type of habitat and corresponds to a surface of 13.5×13.5 m. White and light green cells correspond to domestic and peridomestic habitats, respectively. Black cells make up the first ring of forest cells surrounding the village, to which population dynamics rules are applied, while dark green forest cells are considered as inactive.

The grid was designed to provide a spatial description of the village of Teya. For this, a raster map of 88×104 pixels derived from the satellite image and the GPS coordinates of all houses was produced (Fig. 1). Within the village, cells were classified into two different types corresponding to domestic (houses) and peridomestic (yards and streets around the houses), and referred to as **A_d_** (480 cells) and **A_p_** (4,847 cells), respectively. Yards consisted mostly of open rocky ground with little or no grass, and with isolated trees. Some may harbor corrals for domestic animals. The sylvatic habitat corresponded to a mixture of large patches of low bushes, forest, and agricultural land (mostly corn and heneken fields). Cell size (13.5×13.5 m), was selected 1) to correspond to the spatial scale at which data were collected and available for fitting the models, and 2) to provide an appropriate compromise between an accurate spatial description and model complexity. Basically, we matched the size of one cell to the size of a typical house since field data available for fitting were abundance per house. Furthermore, since houses are obviously much smaller than the peridomestic and forest areas surrounding them (see Fig. 1), larger cells would have led to a non-accurate spatial description by mixing domestic and peridomestic habitats within any ‘domestic’ cell. On the other hand, smaller cells would have lead to additional complexity in the model since description of dispersal at smaller spatial and temporal scales would have been required, for which no data was available.

#### The sylvatic habitat modelled by boundary conditions

Due to a lack of reliable information, the number of triatomines and their population dynamics in the sylvatic habitat (bushes, forest and agricultural land around the village) was not explicitly included in the model. However, the contribution of the forest habitat to the population dynamics of triatomines within the village was accounted for by defining boundary conditions that described immigration from the sylvatic colonies established outside of the village (see dispersal rules below). As usual in spatially explicit models [20] boundary conditions were applied only to one ring of cells, which here corresponded to the first ring of cells surrounding the village and that we refer to as **A_s_** (466 cells). The rest of the forest cells that appear on the map (Fig. 1) were thus effectively inactive during simulations. Accordingly, our model primarily was designed to track the number of *dispersing adult* triatomines in each cell inside the village.

#### Adults dispersing inside the village as state variables

We considered only adult individuals since previous studies have shown that >90% of the individuals found in houses are adults [14], [19]. In addition, our model focused on the number of dispersing adults, thereafter also referred to as dispersers, i.e., those adult individuals that have left their colonies. Accordingly, our model does not track the variations in the number of ‘non-dispersers’ adults, i.e., those that are still in the peridomestic (and sylvatic) bug colonies. The main reason for these choices is that our bugs abundance data correspond to the number of triatomines found in houses, so that to model the dynamics of the colonies would be speculative. Those colonies were assumed to be of constant size and to produce dispersing adults at a constant rate during the dispersal period (see below). This kind of assumption is very common in source-sink models [34]. The state variables of the model were thus denoted N**_c_**(t) the number of *dispersing adult* triatomines present at time t in cell c, with c ∈ **A_d_** (the domestic cells) or c ∈ **A_p_** (the peridomestic cells). The time step of the model was fixed to 2 weeks, so that model predictions were produced with the same time resolution as the available data. At each time step, local and dispersal rules [35] were applied sequentially.

#### Local demographic rules for individuals dispersing inside the village

The local demographic rules applying to each cell at each time step were kept as simple as possible, based on previous observations. Reproduction in domestic cells was neglected since house infestation is associated with a limited or virtually null fertility [10], [15], [18]. Reproduction in the peridomestic habitat was implicitly accounted for by considering those cells as fixed ‘sources’ [36], [37], where individuals are born and can disperse from (see the definition of K_p_ below). Local demographic rules thus involved only two parameters, the survival of dispersing adults in the domestic (S_d_) and peridomestic habitats (S_p_), and can be written:
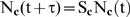
(1)where N**_c_**(t+τ ) is the number of dispersing adults in cell c after the survival process, and with S**_c_** ∈ {S_d_, S_p_}. Here, τ does not take a specific time value. It is only defined to apply the events of survival (Eq. 1) and dispersal (Eq. 2a and 2b below) in a sequential way.

#### Dispersal rules

The individuals that were already dispersing inside the village and that survived the demographic part of the time step t, i.e. N**_c_**(t+τ), can either leave cell c according to a probability d**_c_** ∈ {d_d_, d_p_}, where d_d_ and d_p_ apply to the domestic and peridomestic habitat, respectively, or stay in cell c according to probability 1−d**_c_**. In addition, new individuals can enter the pool of dispersers by leaving one of the two sources; the peridomestic and sylvatic bug colonies. We denoted K_p_ and K_s_, the total number of individuals leaving colonies established in **A_p_** and **A_s_**, respectively. As specified above, reproduction inside houses was neglected, so that there was no colony established in the domestic habitat. Accordingly, there is no such parameter K for the domestic habitat. When a dispersing individual left cell c, the cell where it dispersed to was chosen according to the intrinsic dispersal capabilities of the bug (distribution of dispersal distances and responsiveness to potential house attraction) and the environment (type of habitats within the range of dispersal distances and their potential attraction). A zero truncated Gaussian with modal distance D and standard deviation σ was used as distance kernel f(r_nc_) to describe how the probability of a dispersal event from cell n to c changes with distance r_nc_. Each probability of dispersal from cell n to c was weighted by a factor H corresponding to the type of habitat in cell c. Weighting factors were standardized so that H = 1 for the peridomestic and sylvatic habitats and H>1 for the domestic habitat. H then measures the relative attraction of houses. From these weighted probabilities we defined the set of cells, denoted N, where an individual can disperse to. We restricted dispersal to the nearest cells that collectively accounted for a 0.99 probability. For each departure cell (denoted ‘n’ below), this restricted set of weighted probabilities was then normalized so that dispersal probabilities add up to one when summed over all possible arrival cells (denoted ‘c’ below). The dispersal rule can then be written:
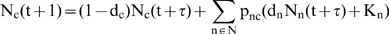
(2a)where p_nc_ stands for the normalized probabilities of dispersal from cell n to cell c, and K_n_ ∈ {K_p_/|**A_p_**|, K_s_/|**A_s_**|}, with |**A_p_**| and |**A_s_**| denoting the number of cells in sets **A_p_** and **A_s_**. As explained above, the number of individuals in the forest habitat was not followed. Individuals reaching or crossing the border of the forest were not numbered in the forest cells, so that they disappeared according to standard absorbing boundary conditions. To mimic *T. dimidiata* seasonal infestation pattern [10], [14], [15], [18] these dispersal rules were applied during a three months ‘immigration period’. Our model does not predict the between years variations in the start of the immigration period, which is likely to depend on many factors acting in the sylvatic and peridomestic habitats that were not included in the model. We thus fixed the immigration period according to the observations made during these two years that is March 16–June 15 in year 1 and April 1-July 1 in year 2. During the rest of the year no individual leaves the colonies to join the dispersing pool, though already dispersing individuals were still allowed to move between cells. During these non-immigration periods, K_n_ = 0, and equation 2a became simply:
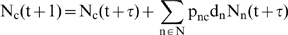
(2b)The spatial and temporal population dynamics in the village was then obtained by applying alternatively Eq. 1 and either Eq. 2a (from mid-March to mid-June) or Eq. 2b (from mid-June to mid-March). This allowed to calculate the number of bugs at time t+1 (N**_c_**(t+1)) as a function of those numbers two weeks before, i.e., at time t (N**_c_**(t)), for any c ∈ {**A_d_** ∪ **A_p_**}. Changes in cells status, either from N**_c_**(t) to N**_c_**(t+τ), or from N**_c_**(t+τ) to N**_c_**(t+1), were evaluated simultaneously from the earliest states of all cells. Because of the typically low abundance of vectors observed in the field, local and dispersal rules were implemented stochastically. The number of surviving individuals in cell c was determined by randomly sampling into a binomial distribution B(N**_c_**(t), S**_c_**). Similarly, the number of vectors dispersing from cell c was determined by randomly sampling into a binomial distribution B(N**_c_**(t), d**_c_**). Finally, the arrival cell of each of the dispersers leaving their cell was sampled with replacement according to the set of normalized probabilities p_nc_. Accordingly, the model presented above is a spatially explicit stochastic model with 9 explicit parameters (S_d_, S_p_, d_d_, d_p_, K_p_, K_s_, D, σ, and H) to be fitted in the maximum likelihood framework described in one of the following section (see ‘Model Fitting and Parameters Estimates’). The model also includes an implicit parameter (the start of the dispersal season), which was fixed *ad hoc* and outside of the likelihood framework.

### Hypotheses on the origin and dispersal patterns of triatomines

The different hypotheses regarding the origin and dispersal patterns of infesting triatomines were defined and modelled by giving specific values to the model parameters as described below and in Table 1. First, to determine the relative contribution of sylvatic and peridomestic insects to house infestation we considered that infesting bugs came from i) both the sylvatic and the peridomestic habitats (K_p_ and K_s_≠0), ii) the sylvatic habitat only (K_p_ = 0 and K_s_≠0), or iii) the peridomestic habitat only (K_p_≠0 and K_s_ = 0). To examine the departure probability in different habitats, we considered that vectors have j) different departure probabilities in the domestic and peridomestic habitats (d_d_≠d_p_), or jj) the same departure probabilities in both habitats (d_d_ = d_p_). Note that these departure probabilities reflect departure through any kind of dispersal including flight or crawling/walking of the bugs. To assess the distribution of dispersal distances, we considered two types of zero-truncated Gaussian distribution: k) with an optimum distance D≠0, or kk) with a monotonous decrease from D = 0. Finally, we accounted for two additional hypotheses about the propensity of bugs to move toward houses. Bugs may either l) be attracted to the domestic habitat (H>1), or ll) not (H = 1). All these hypotheses about the dispersal process were thus combined in the spatially explicit modelling described in the previous section to evaluate their consequences on the spatio-temporal dynamics of infestation. We refer to the ‘complete model’ as the one including dispersal from both sylvatic and peridomestic habitats (i), with different departure probabilities in the peridomestic and domestic habitats (j), a distribution of dispersal distance with an optimum different from 0 (k) and some attraction to the houses (l). From this ‘complete model’, we derived 5 alternative models by modifying independently the hypothesis i to l. Substituting hypotheses ii to i, iii to i, jj to j, kk to k and ll to l, we obtained 5 nested models that we called ‘Sylvatic only’, ‘Peridomestic only’, ‘Same departure probabilities’, ‘Null distance mode’, and ‘Random dispersal’, respectively.

**Table 1:** Parameters of the spatially explicit modelling, hypotheses to be tested and parameter values used in the analysis.

Parameter description	Hypotheses	Parameter values
Survival in the domestic habitat (S_d_)	None	]0,1[
Survival in the peridomestic habitat (S_p_)	None	]0,1[
Number of individuals joining the pool of dispersers from colonies of the peridomestic habitat (K_p_)	(i) forest and peridomestic origin	]0,1000]
	(ii) only forest	0
	(iii) peridomestic only	]0,1000]
Number of individuals joining the pool of dispersers from colonies of the sylvatic habitat (K_s_)	(i) general	]0,1000]
	(ii) only forest	]0,1000]
	(iii) peridomestic only	0
Departure probability from the domestic habitat (d_d_)	(j) independent	[0,1]
	(jj) equal	d_d_ = d_p_ ∈ [0,1]
Departure probability from the peridomestic habitat (d_p_)	(j) independent	[0,1]
	(jj) equal	d_d_ = d_p_ ∈ [0,1]
Mode of the distribution of dispersal distances (D)	(k) modal distribution	]0–500]
	(kk) centred distribution	0
SD of the distribution of dispersal distances (σ)	None	]0–500]
Attraction to the houses (H)	(l) attraction by houses	]1–200]
	(ll) no attraction by houses	1

Parameter values are given for a 15 days period, which corresponds to the time step of the models. Parameters were allowed to vary in the indicated range of values to best fit the data with each of the 6 nested models defined with respect to hypotheses i-ii-iii, j-jj, k-kk, l-ll (as described in section ‘Hypotheses on the origin and dispersal patterns of triatomines’).

### Model fitting and parameters estimates

Models were fitted to adjust the predicted to the observed number of bugs in each cell of the 24 maps describing their biweekly distribution within the village over the first year period. The log likelihood value of a given model (LLH, [38], [39] for two thorough appraisals of the use of likelihoods in model fitting) was then defined as follows:
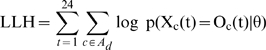
(3)where log denotes the natural logarithm, X**_c_**(t) is a statistical variable corresponding to the number of adults in cell c, O**_c_**(t) the observed abundance in this cell, and θ is the set of parameters of the model to be fitted. The probabilities 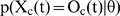 were calculated assuming a zero-inflated Poisson distribution to take into account an excess of null abundance in the data set [40], possibly due to the non-participation of a proportion (w) of householders:
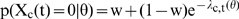
(4a)and
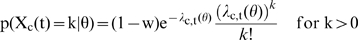
(4b)where the average numbers of individuals in each cell c at time t 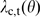 were estimated from 1000 replicates of our stochastic model using the same set of parameter values. Each replicate was started at the beginning of the infestation season with no bug in the village, and was run until an asymptotic equilibrium was reached. All the results presented were obtained for a value of w = 0.7. Lower rates of participation only affected the total number of dispersing individuals, which have to be introduced into the model, but the main conclusions remained unaffected.

Although the parameters were identified using a simple algorithm (see ‘Appendix S1’), the simultaneous estimation of the parameters of each of our model required long computing time. Typically, the parameterization of one model would have required 2 years of calculation on a desktop computer. The program developed to estimate the parameters of our models was thus parallelized, and ran on the ‘Bluegene/P Solution’ of the super-computing centre from the Institut du Développement et des Ressources en Informatique Scientifique located at Orsay, France (http://www.idris.fr/ - Project IDRIS 112290). This program was written in C/MPI.

### Comparison between models

Because models with more parameters often lead to a better fit between predicted and observed data, appropriate statistics accounting for the number of parameters need to be used to evaluate if the added complexity is justified. We used the standard Akaike Information Criterion [33], which applies when the number of data points is large relative to the number of parameter p in the model [41]:
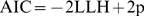
(5)


Accordingly, the better the fit (i.e. the larger the LLH), and the simpler the model (i.e., the fewer the parameters), the lower the AIC. The model receiving the most support from the data is then the one having the lowest AIC. For more systematic comparisons, AIC values were transformed to delta AIC values (Δi). These values represent the differences between the AIC value of a given model i (AICi) and the minimum AIC value (AICmin) associated with the best model (Δi = AICi−AICmin). Thus, the best model has Δi = 0, while the rest of the models have positive values. Δi are then used to categorize the level of support for model i [42] as substantial (when Δi≤2), considerably lower than the best model (4≤Δi≤7) and essentially none (Δi>10). In addition, one can calculate the Akaike weights for each model:
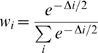
(6)which allows to quantify the probability that each model is the best approximation to the truth [33]. The pairwise ratios between those probabilities then allow comparing the relative strength of support for two models and the corresponding hypotheses.

### Test of the models

The ability of the different models to predict the spatio-temporal distribution of bug abundance was further measured by correlating the numbers of bugs predicted by the model and two observed data sets, including one used for model fitting, and a replicate data set from a second year. Data were pooled over 3-months periods (starting in mid-September) and within three distance categories: 0–80 m, 81–200 m and >200 m from the bush area outside the villages [19]. A Poisson regression between observed and predicted abundances was performed, and a McFadden's likelihood ratio index was used as a pseudo R-squared.

## Results

### General evaluation of the models

**Figure 2:**
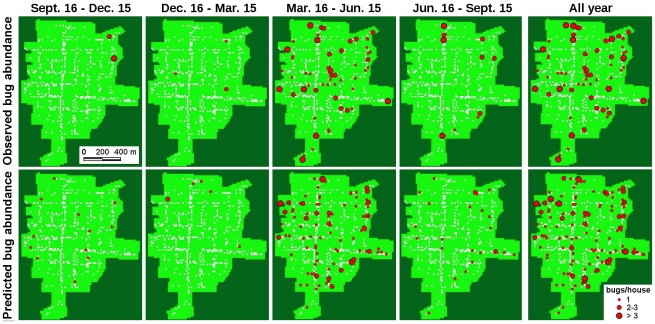
Observed and simulated dynamics of infestation in the village of Teya, Yucatan, Mexico. Circles indicate the location of triatomines and their size is proportional to the number of bugs. The first row shows the temporal variation in the spatial distribution of vectors observed between September 2006 and 2007. The second row shows one stochastic simulation produced with the best parameterized model (the ‘Same departure probabilities’ model) for the same period.

**Figure 3:**
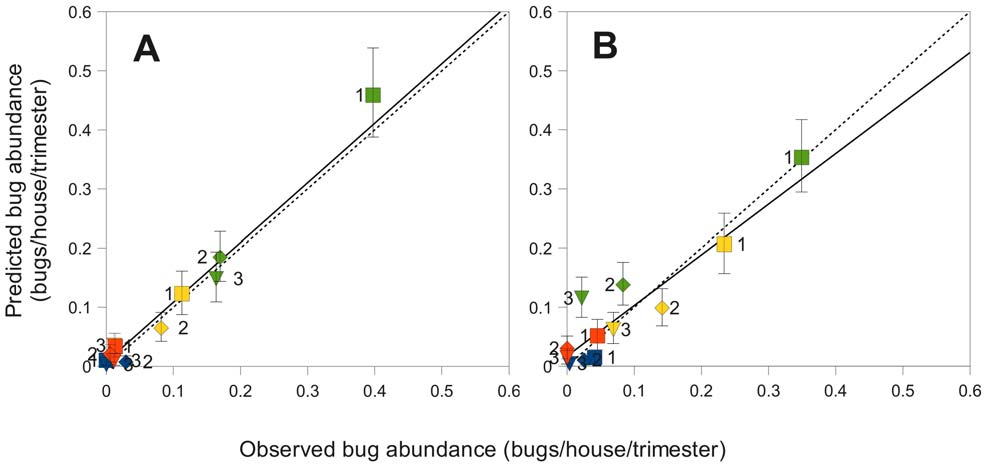
Correlation between observed and predicted bug abundance in the village of Teya, Yucatan, Mexico. (**A**) Descriptive value of the model as indicated by the relationship between predicted values and abundance data during the first year of field collections that were used to estimate the model parameters. (**B**) Predictive value of the model given by the relationship between predicted values and the abundance data during the second year of field collections. Total bug abundance observed in the houses and estimated in the model were pooled over 3-months periods (starting in mid-September 2006) and within three distance categories: 0–80 m, 81–200 m and >200 m from the bush area outside the villages. The 95% confidence intervals correspond to the variations in bug abundance obtained in the 1,000 stochastic simulations performed to fit the model to the data. Predicted abundance = 0.008+1.009 * Observed abundance year 1 (McFadden's likelihood ratio index = 0.919). Predicted abundance = 0.019+0.854 * Observed abundance year 2 (McFadden's likelihood ratio index = 0.750). The straight and dotted lines correspond to the above regression lines and 1∼1 relationship, i.e., a perfect fit. Squares, diamonds and triangles stand for the mean abundance predicted in the three following spatial areas: 0–80 m, 81–200 m and >200 m from the bush area outside the villages. Green symbols stand for the ‘immigration period’, which corresponded to March16–June 15 in year 1, and delayed by 15 days in year 2 (because immigration started latter during this second year). Yellow, orange and blue symbols stand for the three following consecutive periods of time during the year. Labels ‘1’, ‘2’, and ‘3’ have the same meaning as squares, diamonds and triangles and are added to ease the reading of colourless printings.

We first performed a general evaluation of how well the different models were able to fit the data, before comparing them to test our hypotheses about the origin and dispersal characteristics of the vectors. The best model identified by the lowest AIC was the ‘Same departure probabilities’ model, in which individuals have the same departure probabilities in both the domestic and peridomestic habitats. Figure 2 shows the dynamics of infestation observed in the village of Teya, and the result of one simulation (one of the 1000 replicates ran as described in ‘Model Fitting and Parameters Estimates’) obtained with this best model. Despite of the stochasticity associated with the low population size, the model predictions fitted the yearly spatial distribution of vectors as well as the seasonal variations observed in the village of Teya satisfactorily well (see the complete description of the best model predictions and quality of the fit with the data in the ‘Appendix S2’). Further correlation analysis between the observed and simulated data of the spatio-temporal dynamics of infestation confirmed that the model reproduced both the low and high triatomine densities at different time of the year and in the different zones of the village (Figure 3A, McFadden's likelihood ratio index = 0.924). Importantly, the model could also predict the observed distribution of the following year (Figure 3B, McFadden's likelihood ratio index = 0.753). Model predictions during the ‘immigration period’ (March–June, green symbols), are lower during year 2 than during year 1, while the opposite holds for the following trimester (July–September, yellow symbols). This is because the actual immigration period was from March16 to June 15 in year 1, and was delayed by two weeks in the second year, but the periods of time when data were collected and predictions remained the same. Accordingly, during the second year, model predictions for the ‘immigration period’ included 15 days with no immigration, and predictions for the following period included 15 days of immigration.

The Δi calculated with respect to the best model described above allow evaluating the quality of the fit provided by the 5 other proposed models. Δi for the ‘Complete’ model and the ‘Null distance mode’ model, including a null distance mode of the dispersal distance distribution, were lower than 2, indicating a substantial support from the data for these two alternatives (Table 2). This suggested that these three models provided roughly equivalent descriptions of the data, which was confirmed by good correlations between observed and predicted spatio-temporal dynamics with both the ‘Complete’ (McFadden's likelihood ratio index equals 0.919 and 0.750 when calculated with the data of the first and second year, respectively) and the ‘Null distance mode’ (McFadden's likelihood ratio index equals 0.928 and 0.733) models.

**Table 2:** Model comparison and parameter values for the best fit of each model.

Model	LLH	AIC	Δ *i*	*w_i_*	K_p_	K_s_	d_d_	d_p_	D	σ	D_m_	H	S_d_	S_p_
Complete (i,j,k,l)	−712.1	1442.2	1.78	0.21	55.3	43.6	0.36	0.30	38.9	31.0	57.4	14.7	0.50	0.95
Sylvatic only (ii)	−733.6	1483.1	42.7	≈10^−10^	0	52.4	0.02	0.06	14.6	247.9	259.6	35.9	0.68	0.96
Peridomestic only (iii)	−722.7	1461.4	21.1	≈10^−5^	51.7	0	0.42	0.22	10.7	37.1	44.7	72.0	0.65	0.98
**Same departure probability (jj)**	**−712.2**	**1440.4**	**0**	**0.52**	**69.4**	**56.1**	**0.38**	**0.38**	**49.3**	**17.7**	**55.5**	**10.5**	**0.37**	**0.97**
Null distance mode (kk)	−713.0	1442.1	1.68	0.22	59.9	47.8	0.91	0.42	0	44.6	47.2	16.4	0.44	0.97
Random dispersal (ll)	−714.7	1445.3	4.95	0.04	198.6	241.7	0.15	0.14	60.5	9.5	62.2	1	0.74	0.81

Hypotheses and parameters are as described in Table 1. In addition, D_m_, the mean dispersal distance (in meters), has been calculated from the estimates D and σ. Along with the name of each model, the first column includes a summary of the underlying hypotheses. The ‘Complete model’ corresponds to hypotheses i,j,k,l, which is noted (i,j,k,l). Each of the other models is defined by changing one of those hypotheses, and only this changed hypothesis is reported. The best model and corresponding optimal parameter values appear in bold. Boxed cells indicate that the parameter values have been constrained according to the hypotheses being considered.

On the contrary, the Δi of the models in which infesting bugs came from the ‘Sylvatic only’ or the ‘Peridomestic only’ were larger than 10, indicating that these models received virtually no support from the field data. Finally, the Δi = 4.95 obtained for the ‘Random dispersal’ model, which includes no attraction by the houses, revealed that this model had a considerably lower support than the best model (Table 2).

### Testing hypotheses about the origin and dispersal characteristics of triatomines

The main interest of the selection model approach is to compare how well different models fit the data, and accordingly how much support the different hypothesis (underlying the competing models) receive from the data. We thus used the above results to test our different hypotheses about the origin and dispersal characteristics of the triatomines. To do so, we compared models within subsets specifically determined to confront our hypotheses about the origin (i vs ii vs iii) and dispersal characteristics (j vs jj, k vs kk, l vs ll).

#### What is the contribution of peridomestic and sylvatic insects to house infestation?

To answer this question, we restricted the model comparison within a first subset of models including the ‘Complete’ (hypothesis i), ‘Sylvatic only’ (hypothesis ii) and the ‘Peridomestic only’ (hypothesis iii) models. As expected from the above general evaluation, the ‘Complete’ model received considerably more support from the data than the other two models. The Akaike weight of the ‘Complete’ model (w = 0.21) is larger, by several orders of magnitude, than the weights of the ‘Sylvatic only’ (w≈10^−10^) and ‘Peridomestic only’ (w≈10^−5^) models (Table 2). The ‘Complete’ model is thus about 2.10^9^ and 2.10^4^ times more likely to provide the best reproduction of the data than the ‘Sylvatic only’ and the ‘Peridomestic only’ model, respectively. In other words, bug populations established in both the peridomestic and the sylvatic habitats contributed significantly to house infestation.

Interestingly, the percentage of vectors immigrating from the peridomestic habitat (i.e., 100*K_p_/(K_p_+K_s_)) was equal to 55.9% in the ‘Complete model’, and nearly identical values were obtained with the two other best supported models (with a Δi<2); the ‘Same departure probabilities’ model (55.1%), and the ‘Null distance mode’ model (55.5%) (Table 2). This strongly suggested that triatomine colonies established in the sylvatic and peridomestic habitats both contributed substantially to house infestation, although with a somewhat larger immigration from the peridomestic habitat. This is confirmed by the determination of likelihood-based confidence interval for the best model (See ‘Appendix S2’). Bugs emigrating from sylvatic sites are indeed more likely to move back outside of the village than bugs emigrating from peridomestic sites, simply because the former are more likely to be located in cells close to the border of the village. Accordingly, the above proportion of bugs emigrating from the peridomestic sites is likely to be an estimation of the minimal contribution of this habitat to house infestation.

Further examination of the parameter values obtained by fitting the different models indicated that in all but the ‘Random dispersal’ model, the total number of individuals dispersing from each of the two habitats (K_p_ and K_s_) were around 40–70 individuals/15 days (Table 2), corresponding to a yearly total of 240–420 bugs dispersing in the village. The larger number of individuals dispersing from these habitats in the ‘Random dispersal’ model was straightforward as more dispersing individuals were needed to fit the abundance observed in houses because those individuals were no longer assumed to be attracted to the houses.

#### What are the rates of departure from the domestic and peridomestic habitats?

Comparison between the ‘Complete’ model (hypothesis j) and the ‘Same departure probabilities’ model (hypothesis jj) indicated that to account for a difference in the departure probability between the domestic and peridomestic habitats did not allow for a better fit. Indeed, Akaike weights of these two models indicated that, because of its lower complexity, the ‘Same departure probabilities’ model (w = 0.52) was twice as likely to be the best model as the ‘Complete’ model (w = 0.21) (Table 2). As expected, this is consistent with the approximately similar departure probabilities in the domestic (d_d_) and peridomestic (d_p_) habitats estimated from the ‘Complete’ model, where these two parameters are not constrained to be equal. The ratio d_d_/d_p_, estimated for this model is indeed equal to 1.2. In other models this ratio lies in a range weakly dispersed around 1, i.e. between 0.33 (‘Sylvatic only’) and 2.2 (‘Null distance mode’) (Table 2), which suggests that our conclusion that vectors disperse at similar rates in the domestic and peridomestic habitat is robust to the detail of the modelling. Although departure probabilities appear to be similar, it is interesting to note that the departure probability from the domestic habitat is consistently (slightly) larger than the departure probability from the peridomestic habitat (with the exception of the ‘Sylvatic only’ model).

The values obtained with the two models of the subset being considered (the ‘Complete’ and ‘Same departure probabilities’ models) are roughly similar and correspond to a rate of departure in the range 0.3–0.4 for a 15 days period (Table 2).

#### What is the distribution of dispersal distances?

Comparing the fit of the ‘Complete’ (hypothesis k) and the ‘Null distance mode’ (hypothesis kk) models indicated that both provided equally good descriptions of the data, as revealed by their Akaike weight which were 0.21 and 0.22, respectively (Table 2). This can be explained by a negative co-variation between the modal distance D and the standard deviation σ of the dispersion kernel,. which allowed to obtain a similar mean distance of dispersal (D_m_) for the two models (Table 2). Accordingly, there was a similar co-variation between D and σ in the other models, which lead to very limited changes in D_m_ values between models (44 to 62 m). The only exception to this trend was the value of D_m_ obtained for the ‘Sylvatic only’ model, since, in this case, longer dispersal distances are required for sylvatic individuals dispersing from the border of the village to reach its centre. Thus, these results suggested that the exact shape of the distribution of dispersal distances was not very important, as long as it resulted in a mean distance of 40–60 m/15 days (Table 2). This quantitative figure was absolutely consistent with the likelihood-based confidence interval determined for the dispersal distance in the best model (See ‘Appendix S2’). Shorter dispersal distances would not explain the presence of vectors in the centre of the village, while larger ones would not allow reproducing the observed gradient of infestation.

#### Are vectors attracted to the houses?

We finally compared the ‘Complete’ (hypothesis l) and ‘Random dispersal (hypothesis ll) models to test whether vectors are or not attracted to houses. As indicated by Akaike weights, the ‘Complete’ model (w = 0.21), which accounts for attraction of the bugs to the houses, is five times more likely to be the best model than the ‘Random dispersal’ model (w = 0.04). The conclusion that vectors are attracted by the domestic habitat is further supported by the high values that the attraction factor H takes in other models where this parameter is allowed to vary (Table 2). The attraction of bugs to the domestic habitat was indeed always larger than 10 and lies between 10.5 and 16.4 in the three best models, suggesting that an house is about 10 times more attractive to the bugs than an identical surface of peridomestic habitat. Such estimate was also consistent with the likelihood-based confidence interval for parameter H obtained with the best model (See ‘Appendix S2’).

## Discussion

A major challenge for the prevention of Chagas disease is to control the risk of transmission associated with non-domiciliated vectors [1]. This epidemiological situation has now been documented in several triatomines species and regions [9], [14], [27], [43], [44]. To design effective control strategies against these vectors clearly relies on an accurate description of their spatial and temporal variations in abundance, and a proper understanding of the dispersal processes generating those distributions.

Studies of the dispersal potential of individuals and the related population spatio-temporal structures are notoriously difficult, mostly because statistically relevant spatial and temporal data sets are difficult to collect in the field (although the use of presence/absence data can allow using the meta-population theory, see [34]). This difficulty is increased when population sizes are low and demographic stochasticity becomes important, as well as when spatial distribution of individuals varies significantly through time, all of which apply to non-domiciliated vectors and more specifically to *T. dimidiata* in the Yucatan peninsula, Mexico [14], [15], [19]. In this paper we addressed this complexity by comparing the ability of different spatially explicit population dynamic models to reproduce the observed spatio-temporal dynamics of infestation at the village scale. We identified three models that provided not only a very good fit to the spatio-temporal variation of abundance observed during the first year time-series of the village of Teya, but that were also able to predict with a very high accuracy the spatial and temporal variation of abundance during the second year time-series of this village. This accuracy suggests that while the models remained rather parsimonious, they included the most relevant factors and variables underlying *T. dimidiata* infestation process. Hypothetically, scaling effects might affect the outcomes of our model since the choice of the size of the cell (13.5×13.5 m) was rather specific. The specification of these dimensions was indeed strongly constrained by both the objectives of the modelling and the spatial scale at which data were collected and thus available for fitting the model (see ‘Spatially explicit sources-sink modelling’ for details). However, it is highly unlikely that changing the size of cell, while keeping them in a range that satisfy the constraints evocated above (i.e., to 10×10 m or 15×15 m), may affect our main results because the strong spatial pattern in the data (gradient in bugs abundance) that is responsible for most of the fit, occurs at a much larger scale (village). The results derived from these models thus allowed us to draw three major conclusions on the characteristics of dispersal of non-domiciliated *T. dimidiata*. Because the spatio-temporal pattern of triatomine abundance has been observed not only in Teya, but also in several villages of the Yucatan peninsula [19], our conclusions are likely to be relevant to the whole region.

First, we obtained strong evidence that *T. dimidiata* found inside houses originated from colonies in both sylvatic and peridomestic sites, and that each habitat contributed substantially to house infestation. This conclusion is robust to the detail of the modelling since all three best models provided the same estimate of the percentage of bugs emigrating from each of these two habitats (the percentage of emigration from the peri-domicile was equal to 55.9%, 55.1% and 55.5% in the ‘Complete’, the ‘Same departure probabilities’ and the ‘Null distance mode’ model, respectively). The mixed origin of insects immigrating into the domestic habitat is also consistent with previous population genetic studies identifying gene flow from both peridomestic and sylvatic habitats [17]. Interestingly, population genetics data suggested that the relative contribution of the two habitats may vary from one village to another, as the estimated proportion of bugs arriving from the peridomestic habitat was found to be of 53% and 84% in the nearby villages of Dzidzilche and Tetiz, respectively when using tests of morphometric assignment [17]. Such field evidence that immigration from the peridomestic habitat into the houses is more important than immigration from the sylvatic habitat is consistent with our modelling results. Indeed the percentages mentioned above are likely to be minimal estimation of the peridomestic contribution to house infestation. This is also in agreement with the identification of several characteristics of the peridomestic habitat as risk factors for house infestation in the city of Merida in the Yucatan [45], as well as the good efficacy of peridomestic environmental management for the control of *T. dimidiata* in Costa Rica [46]. Taken together, these observations confirm that the peri-domicile is a key target for the control of non-dimiciliated triatomines, but strategies should nonetheless be designed to simultaneously reduce infestation by insects of sylvatic origin.

A second main outcome of the models is related to the dispersal pattern, which seems to be of secondary importance compared to the mean distance of dispersal itself, which was also rather short. Again, this conclusion is very robust to the details of the modelling since the mean path distance achieved by moving individuals was very similar among the three best models (57, 55 and 47m/15 days in the ‘Complete’, the ‘Same departure probabilities’ and the ‘Null distance mode’ model, respectively). This short dispersal distance appears necessary to account for a persisting spatial gradient of abundance with higher level of infestation in the periphery of the village, likely due to immigration from the sylvatic habitat surrounding the village [19], [20]. This estimate of dispersal distance is consistent with, albeit lower than, available data on dispersal of other triatomine species indicating that *T. infestans* and *T. sordida* individuals have the potential to fly over several hundred meters [21], [22], [24], [47]–[50], although the difficulties of following triatomines over larger distances make such estimates uncertain [21], [24], [51]. Assuming straight pathways, short dispersal distances could indicate that movements of bugs inside the village consist of walking/crawling rather than flying. Alternatively, actual dispersal pathways may be much longer but with many changes in direction, resulting in a reduced apparent dispersal distance [52]. While these two alternatives could result in a similar spatio-temporal pattern of infestation, they would likely result in important differences in the physiologic status of the bugs due to the elevated cost of dispersal in triatomines, and thus would greatly influence the long-term outcome of the infestation. For example, exhausted insects arriving in a house may have limited abilities to feed, mate, lay eggs, or survive, compared to insects with higher energy supplies. Interestingly, field observations indicate that feeding status and potential fertility of bugs found inside houses remain well below optimum levels [15], and estimates of the survival probability in the domestic habitat derived from the present models were consistently about half of that in the peridomestic habitat. In any case, because dispersal distances are typically short, sylvatic bugs tend to stay in the periphery of the village, where they add up to the vectors originating from the peridomestic habitat and thus define the area with the highest risk of transmission within the village.

The third main result of our study is that houses strongly attract *T. dimidiata*. Typically, houses were estimated to be more than 5–15 times more attractive to bugs than the same surface of peridomestic or forest habitat. As for our previous conclusions, the three best models are again very consistent (house attractiveness of 14, 10, and 16 in the ‘Complete’, the ‘Same departure probabilities’ and the ‘Null distance mode’ model, respectively). The actual stimuli making houses attractive to *T. dimidiata* is unknown, but it is well established that triatomines tend to disperse toward artificial light [27], [28], [52] and are attracted by odors from their hosts [53]–[55]. Hypothetically, the higher attractiveness of domestic cells could thus be explained by a higher abundance of light or hosts in houses. However, given the distances over which houses exert their attraction, artificial light is very likely to be a key factor. In any case, our results suggest that interfering with house attractiveness and triatomine dispersal may represent alternative strategies to reduce house infestation by non-domiciliated vectors.

As mentioned above, the characteristics of triatomine dispersal that we identified have important implications for the type of interventions that may be designed and their efficacy at controlling *T. dimidiata* in the region. First, vectors from both the peridomestic habitat, and those from the forest/bushes surrounding a village have to be controlled. This obviously differs from the situation encountered with *T. infestans* or *T. guayasana*, where immigration from the peridomestic area is thought to be by far the main source of house re-infestation [49], [56], [57], and clearly is a major difficulty when trying to limit house infestation by non-domiciliated vectors. However, the additional evidence that insects disperse only on small distances, which coincides with the highest abundance of vector at the periphery of the village, strongly suggests that spatially targeted control strategies may be proposed. Indeed, the periphery of the village represents not only the area with the highest *T. cruzi* transmission risk [19], [20], but also a transit zone for sylvatic bugs dispersing toward the centre of the village. Hypothetically, insecticide spraying in houses and peridomestic habitats close to the periphery could thus not only limit infestation in the treated area, but also reduce bug abundance in the centre of the village. Alternatively, housing modifications aimed at reducing house attractiveness or at attracting bugs into lethal traps located away from houses may represent alternative or complementary control strategies [55]. The ‘quasi-mechanistic’ models [58] we developed in this contribution provide an excellent framework to further explore the efficacy of such strategies since they allow to reproduce the population dynamics observed in the field from a simple description of bugs demography and dispersal. Also, these models could be easily adapted to other patterns of house infestation and other triatomine species, provided appropriate field data sets describing their respective infestation dynamics are available, thereby allowing a rapid evaluation of potential vector control interventions in a variety of situations.

In conclusion, our results show that non-domiciliated *T. dimidiata* found inside houses in the Yucatan peninsula originate in similar proportions from both sylvatic and peridomestic habitats. They further reveal that dispersing triatomines are strongly attracted to houses, but disperse on rather small distances. Since these dispersal characteristics are associated with much higher abundances of insects in the periphery of the village, they suggest that spatially targeted interventions may allow to optimize the cost efficacy of vector control activities within villages.

## Supporting Information

Alternative Language Abstract S1 Translation of the Abstract into Spanish by Eric Dumonteil.
(0.02 MB DOC)

Appendix S1 Optimization algorithm.
(0.77 MB DOC)

Appendix S2 Additional results.
(0.81 MB DOC)
