# Supplementary material for: Characterization of the Dispersal of Non-Domiciliated Triatoma dimidiata through the Selection of Spatially Explicit Models
Source: PLoS Negl Trop Dis. 2010 Aug 3;4(8):e777. doi: 10.1371/journal.pntd.0000777 (PMC2914783; doi:10.1371/journal.pntd.0000777)
Supplement: Alternative Language Abstract S1 — Translation of the Abstract into Spanish by Eric Dumonteil. (0.02 MB DOC) [file pntd.0000777.s001.doc]

**Alternative Language Abstract S1 - Translation of the Abstract into Spanish by Eric Dumonteil.**

La enfermedad de Chagas es una de las enfermedades desatendidas más importante de America Latina. Aunque insecticidas han sido aplicados con éxito para controlar las poblaciones de vectores domiciliados, esta estrategia queda inefectiva en las áreas donde los vectores inmigran desde ecotopos peridomesticos o silvestres para (re-)infestar las casas. El desarrollo de estrategias para el control de estos vectores no-domiciliados ha sido identificado por la Organización Mundial de la Salud como unos de los principales retos para el control de la enfermedad. Para ello, se requiere de una descripción de la dinámica espacio-temporal de infestación por estos vectores y de su dispersión. Combinamos por primera vez datos espacio-temporales extensivos que describen la dinámica de infestación por *Triatoma dimidiata* en una localidad, con modelos espaciales de la dinámica poblacional. Los modelos se ajustaron y predijeron muy bien la dinámica de infestación observada. De esta manera, los modelos proporcionaron tanto una información clave sobre la dispersión de *T. dimidiata* en este area, así como un marco matemático apropiado para evaluar la eficacia de varias estrategias de control. De manera interesante, los patrones de infestación observados y modelados sugieren que las intervenciones podrían enfocase en la periferia de las localidades, donde el riesgo de transmisión es más alto. Tal optimización espacial podría ayudar a reducir el costo de las intervenciones y compensar por la repetición de estas debido al carácter no-domiciliado de los vectores.
