# Supplementary material for: Characterization of the Dispersal of Non-Domiciliated Triatoma dimidiata through the Selection of Spatially Explicit Models
Source: PLoS Negl Trop Dis. 2010 Aug 3;4(8):e777. doi: 10.1371/journal.pntd.0000777 (PMC2914783; doi:10.1371/journal.pntd.0000777)
Supplement: Appendix S1 — Optimization algorithm. (0.77 MB DOC) [file pntd.0000777.s002.doc]

# Appendix S1 – Optimization algorithm


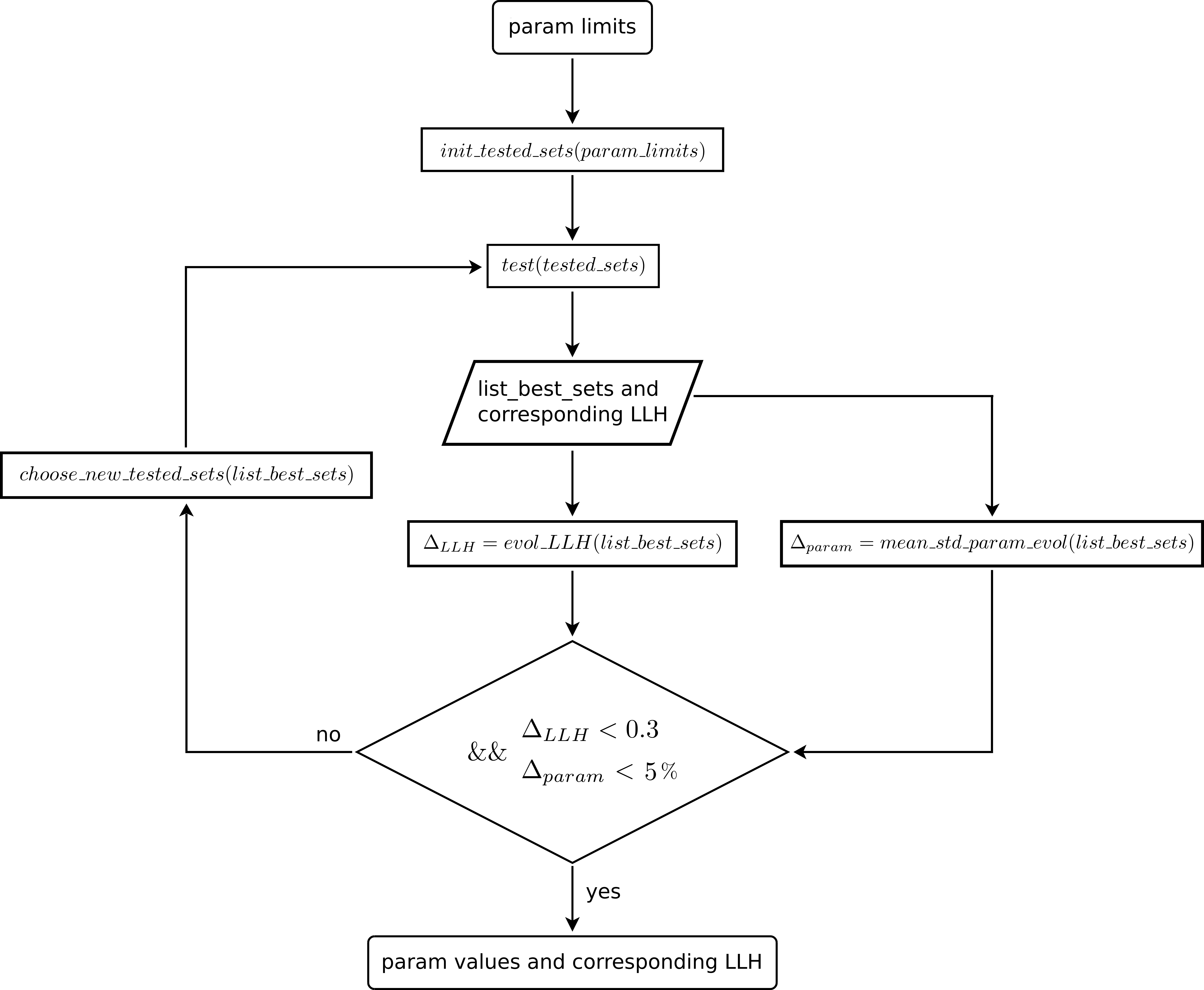


The range of parameter values to be tested (as given in table 1) was specified in ‘param limits’. In this range, the parameter values providing the best fit were searched for using a recursive algorithm summarized in the above diagram. Functions used in this algorithm are described below.

For each loop, a set of three values per parameter was tested. The initial set of parameter values to be tested (***init_tested_sets)*** corresponds to the quartiles of the range allowed for each parameter. Afterwards, this set of parameter values (*tested_sets*) was identified at the end of the previous loop (see below function *choose_new_tested_sets*).

The function *test* calculated and returned the LLH corresponding to all combinations of the three tested values per parameter. We kept the sets of parameter values with a probability over 5% to be the best fit in the list of best sets (*list_best_sets*) for further computations.

The *evol_LLH* function evaluated the improvement in the quality of the fit (Δ _LLH_) between loop *i* and loop *i-1* as the difference between the mean of the LLH currently in *list_best_sets* and the mean of LLH calculated from the same set in loop i-1*.* Simultaneously, *mean_std_param_evol* evaluated the change in the parameter values corresponding to the best fits obtained in loop *i* and loop *i-1*. For each parameter, this last function calculated the difference in the mean of the values currently in *list_best_sets* and the mean calculated in the same way in loop i-1 (Δ _param_). From *list_best_sets*, it also calculated the standard variation for each parameter, as this was further needed in function *choose_new_tested_sets* (see below).

Unless the change in the LLH (Δ _LLH_) was lower than 0.3 and the largest change in the mean of parameter values lower than 5%, a new set of parameter values was tested to improve the quality of the fit. The new set of parameter values to be tested was determined by *choose_new_tested_sets*. For each parameter, the three new values used were the mean, and the mean ± sd (as calculated in *mean_std_param_evol*)

The final LLH and selected param values were respectively the mean LLH and the mean parameter values in the last *list_best_sets*.

The corresponding code is available upon request by interested readers.
