# Supplementary material for: Characterization of the Dispersal of Non-Domiciliated Triatoma dimidiata through the Selection of Spatially Explicit Models
Source: PLoS Negl Trop Dis. 2010 Aug 3;4(8):e777. doi: 10.1371/journal.pntd.0000777 (PMC2914783; doi:10.1371/journal.pntd.0000777)
Supplement: Appendix S2 — Additional results. (0.81 MB DOC) [file pntd.0000777.s003.doc]

**Appendix S2 – Additional Results.**

The model providing the best fit to the data was the ‘same departure model’ (see Table 2 in the main body of the paper). This supplementary file provides additional results on the predictions of this model, and the quality of the fit to the data it provided. It also includes likelihood profiles and likelihood-based confidence intervals around each of parameter values corresponding to the best fit.

**Best model predictions and quality of the fit with the data.**

The following results show how the model reproduced the observed frequency distribution of bug counts per site and two-weeks period (Figure S1), the absence of appreciable spatial and temporal correlation at those scales (Figure S2 and S3), as well as the spatial and temporal variations of bugs distributions at the village scale (Figure S4).

Data collections and model predictions were performed by house and per two-weeks. As illustrated in figure S1, the abundance of bugs at this spatial and temporal scale is low, which is consistent with the non-domiciliated status of the triatomine populations under study, and previous estimates of vector abundance in the Yucatan peninsula (e.g., Dumonteil et al 2002, Dumonteil and Gourbière 2004).

**Figure S1**. **Predicted and observed frequency distribution of bug counts per site and fortnight.** Grey : data collected between August 2006 and August 2007. White: predictions made with the ‘same departure model’ described in the main text. For most time of the year, there is no bug in the houses of the village, and this leads to the high frequency of 0 in both the model and the data. During the season of dispersal, bugs infest houses where they are found in abundance typically ranging from 1 to 3 per two-weeks, as it appears both in the data and in the model.

At these spatial and temporal scales there was almost no spatial and temporal autocorrelation. The temporal autocorrelation functions (Venables and Ripley, 2002) calculated from observed and predicted house bug abundances were indeed not statistically significant at all tested time lags (R-Package *stats* version 2.8.1 available at <http://cran.r-project.org/>) (Figure S2).

**Figure S2**. **Temporal auto-correlation in bugs abundance per house and per two-weeks**. Grey and white have the same meaning as in Figure S1. Temporal autocorrelation between abundance at t and t + Time lag reach statistical significant when the lie outside the range –0.4 to +0.4. Accordingly, no correlation was observed at any time lag different from 0. Though the pattern obtained from the model was obtained from only one simulation, the exact same pattern was consistently observed in another 300 simulations.

Spatial autocorrelation of fortnight bugs abundance, measured by calculating the Moran index and the associated Z-scores (Griffith, 1987), reached marginal statistical significance during only one two-weeks period of the year, both in the data and in the model (Figure S3). The temporal variations of Moran’s index in the data and in the model were similar (pvalue=0.19, Wald-Wolfowitz run test).

**Figure S3**. **Spatial auto-correlation in bugs abundance per house and per two-weeks**. Grey and white have the same meaning as in Figure S1. Stars indicate that marginally significant spatial autocorrelation, i.e. leading to calculated p-value in between 0.03 and 0.05, was observed during two two-weeks periods, both in the data and in the model. Over our 300 simulations, such spatial correlation was significant for on average <1 two week-period of time during the year (when averaged over the 300 simulations).

Given such typically low abundance of bugs per house and per two weeks, comparison between the model and the data were also performed after pooling bugs present in all houses of the village at each two-weeks period, and after pooling bugs present at the various time of the year in any given house.

The observed and predicted spatial distributions obtained after pooling bug collections through time are provided in Figure 2 in the main body of this paper. Spatial autocorrelation measured on the data (Moran’s Index=0.003, pvalue=0.196), and model (Moran’s Index=0.069, pvalue=0.0832) were consistently non significant. Finally, the model was able to reproduce the spatial variations of abundance after data were pooled in concentric zones from the outside to the centre of the village (Figure 3 of the manuscript).

The observed and predicted temporal distributions obtained after pooling bugs collection through space are provided in figure S4.

**Figure S4.** **Predicted and observed time series of total bugs on domestic sites per two-weeks**. Grey and white have the same meaning as in Figure S1.

Clearly, the predicted variations matched the seasonal variations observed in the field at the scale of the village with no trends in residuals (pvalue=0.93, Wald-Wolfowitz run test), and the autocorrelation function (Venables and Ripley, 2002) on data and model showed a statistically significant correlation (only) for a time-lag=1.

Overall, the model reproduced the observed frequency distribution of bug counts per site and two-weeks (Figure S1), the absence of appreciable spatial and temporal correlation at those scales (Figure S2), as well as the spatial and temporal variations of bug distribution at the village scale (Figure S3 and figure 3 in the manuscript).

Finally, the dispersal kernel corresponding to the best model is provided in Figure S5. It gives the probabilities of dispersal with respect to the distance from the departure cell.

Dispersal probability

**
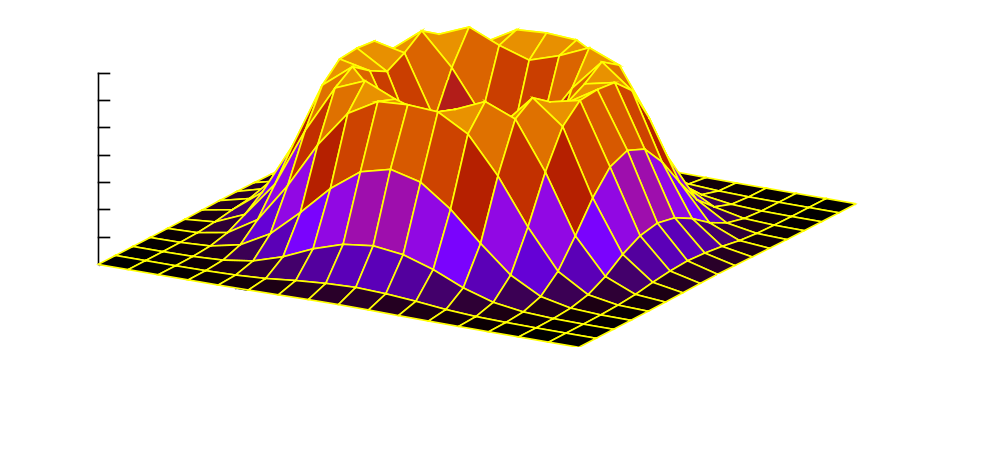
**

Dispersal probability

x_1_ - Distance (m)

x_2_ - Distance (m)

**Figure S5.** **Best dispersal kernel.** The distribution is a zero-truncated gaussian function with a modal distance D=49.3 and a standard deviation σ=17.7 gives the probability for bugs located at (0,0) to move to a position (x_1_,x_2_) in the two-dimensional plane.

**Likelihood surface around each of the estimated parameter values.**

Estimates of the parameter obtained with the best model, the ‘same departure model’ are displayed in the figure S6.

**Figure S6.** **Likelihood surface determined around the set of parameter values providing the best fit.** Predictions were made from the ‘same departure model’ described in the main text. Grey and black crosses are for the production of the dispersers from the colonies (K_s_ and K_p_, respectively), grey and black squares are for the parameters of the zero-truncated gaussian function (D and σ, respectively), grey and black triangles are for the two other dispersal parameters (d_d_=d_p_ and H, respectively) and horizontal lines are for survival in houses.

Such profile likelihoods provided us with the opportunity to draw profile likelihood intervals with upper and lower boundaries corresponding to parameter values decreasing the AIC by 2 points. The profile likelihood intervals we obtained are given in the following table.

| Values | K_p_ | K_s_ | d_d_=d_p_ | D | σ | D_m_ | H | S_d_ | S_p_ |
| --- | --- | --- | --- | --- | --- | --- | --- | --- | --- |
| Lower | 52.0 | 39.3 | 0.22 | 39.4 | 7.4 | 44.4 | 6.5 | 0.29 | 0.90 |
| Estimate | 69.4 | 56.1 | 0.38 | 49.3 | 17.7 | 55.5 | 10.5 | 0.37 | 0.97 |
| Upper | 88.9 | 77.4 | 0.55 | 59.2 | 29.1 | 66.6 | 14.6 | 0.44 | 0.99 |

**Table S1**: **Likelihood-based intervals for the set of parameter values providing the best fit**.

The interval for Kp and Ks were strongly overlapping, which suggested that the ratio between these two parameters is consistently slightly larger than 1. This backed the conclusion that the sylvatic and peri-domestic habitats contributed to houses infestation in roughly equivalent ways, with a slightly higher proportion of bugs found in houses originating from colonies established in the peri-sylvatic habitat. The profile likelihood interval for D and D_m_ confirmed that bugs dispersed on rather short distances, typically 40-60 meters per moving individual. The profile likelihood interval for H ranged from 6.5 and 14.6, which confirmed that bugs are strongly attracted to the domestic habitat. Though, only the estimates of the former key parameters are considered as substantial outcomes of this study, and commented further in the discussion, we have also included the profile likelihood and intervals for the other parameters of the model.

**Literature cited in the Appendix S2.**

[Dumonteil E, Gourbière S, Barrera-Perez M, Rodriguez-Felix E, Ruiz-Pina H, Banos-Lopez O, Ramirez-Sierra MJ, Menu F, Rabinovich JE.](../../../../entrez/query.fcgi%3Fcmd=Retrieve&db=pubmed&dopt=Abstract&list_uids=12389944) 2002. Geographic distribution of Triatoma dimidiata and transmission dynamics of Trypanosoma cruzi in the Yucatan peninsula of Mexico. Am J Trop Med Hyg. 67(2):176-83.

Dumonteil E., and S. Gourbière. 2004. Predicting triatoma dimidiata abundance and infection rate: a risk map for natural transmission of chagas disease in the Yucatan peninsula of Mexico. Am J Trop Med Hyg. 70(5):514-9.

Venables, W. N. and Ripley, B. D. 2002. *Modern Applied Statistics with S*. Fourth Edition. Springer-Verlag.

Griffith, Daniel. 1987. *Spatial Autocorrelation: A Primer*. Resource Publications in Geography, Association of American geographers. 1987.
